# Customized patterned substrates for highly versatile correlative light-scanning electron microscopy

**DOI:** 10.1038/srep07033

**Published:** 2014-11-13

**Authors:** Lorena Benedetti, Elisa Sogne, Simona Rodighiero, Davide Marchesi, Paolo Milani, Maura Francolini

**Affiliations:** 1 Fondazione Filarete for Biosciences and Innovation, Viale Ortles 22/4, 20139, Milan, Italy; 2 Department of Medical Biotechnology and Translational Medicine, Università degli Studi di Milano, and National Research Council (CNR) Neuroscience Institute, Via Vanvitelli 32, 20129 Milan, Italy; 3 Interdisciplinary Centre for Nanostructured Materials and Interfaces (CIMaINa), and Department of Physics, Università degli Studi di Milano, Via Celoria 16, 20133 Milano, Italy; 4 European School of Molecular Medicine (SEMM), IFOM-IEO, Via Adamello 16, 20139 Milano, Italy

## Abstract

Correlative light electron microscopy (CLEM) combines the advantages of light and electron microscopy, thus making it possible to follow dynamic events in living cells at nanometre resolution. Various CLEM approaches and devices have been developed, each of which has its own advantages and technical challenges. We here describe our customized patterned glass substrates, which improve the feasibility of correlative fluorescence/confocal and scanning electron microscopy.

The aim of correlative light electron microscopy (CLEM) is to combine the benefits of being able to observe the sub-cellular features of living cells by means of light microscopy (LM), and reveal their ultrastructural details using electron microscopy (EM)^1^,^2^,^3^. The technique is challenging because it is necessary to switch from one device to the other: each requires an appropriately prepared sample, and one significant bottleneck is caused by the slow and laborious process of relocating a region of interest identified by light microscopy when switching to the electron microscope.

There are two principal types of CLEM: fluorescence/confocal microscopy with transmission electron microscopy (TEM) or scanning electron microscopy (SEM). The first has seen the development of various protocols and devices^4^, and is widely used in biological laboratories^3^,^5^,^6^, but the second is still relatively undeveloped^7^,^8^,^9^, and one of its prevailing limitations is the lack of substrates that are optimally suited for relocating the sample when switching from one imaging mode to the other^8^, in the absence of a single microscope equipped to do both^8^,^10^,^11^.

In order to overcome this limitation, we have produced transparent, metal-patterned glass coverslips that are ideal for high-resolution confocal microscopy, allow cell growth and proliferation, are resistant to electron microscopy sample preparation procedures, and provide optimal contrast for SEM location. Commercially available etched substrates for optical microscopy have proved to be expensive and incapable of providing sufficient contrast for the SEM relocation of samples, whereas our patterned coverslips are cheap and rapid to produce, can be used with all optical and scanning electron microscopes, and are easily customized in terms of the choice of deposited metal and pattern design (Supplementary Figs. 1–3).

As proposed by Jimenez *et al*.,^7^ we initially selected a square-based pattern for experiments using flattened HEK and HeLa epithelial cells (see Methods). Each basic element of the pattern consisted of six solid 150 × 150 μm squares surrounding a letter, was precisely the same size as that of the field of view of the 20× objective of the light microscope (775 × 775 μm) in order to facilitate the rapid mapping of a number of regions of interest, and was repeated 25 times (5 rows × 5 columns) so that it fitted the central area of a 10 mm round glass coverslip (Supplementary Figs. 1 and 2b).

A second, circle-based pattern was designed for experiments using cultured neurons, which are characterised by a cell body (soma) with a diameter of about 30 μm that has a number of protruding thin, branching neurites with a length of tens of micrometres. The correlative analysis of neuronal morphology and neurite branching requires a larger free coverslip surface in order to exclude any possible effect due to interactions between the cell structures and the deposited metal, and so the basic element consists of eight 70 μm diameter solid circles surrounding a letter or a number (Supplementary Figs. 1 and 2a), repeated 30 times (5 rows × 6 columns). Its size was selected on the basis of the same criterion as that used to determine the size of the square-based basic element.

These two models fully satisfied our cell relocation needs, but the same technical approach can be used to customise the pattern on the basis of whatever cell shape (Fig. 1a).

We then used a stainless steel laser-cut mask to transfer the chosen template to normal cell culture coverslips by means of a conventional evaporation system based on the electron beam deposition (EBD) of the metallic target (Fig. 1b). In order to find the metal and film thickness that most easily identified the patterns using either microscopic technology, we tested gold, titanium and zirconium, all of which are known to be biocompatible and support cell adhesion and growth^12^,^13^. The best results were obtained using 70 nm thick deposits of gold, 100 nm thick deposits of titanium, or 100–150 nm thick deposits of zirconium (Supplementary Fig. 3c), although all of the described experiments were performed using titanium because it is less expensive than the other metals, and the process of EBD is faster (see Methods). The patterned coverslips were sterilized and functionalized before seeding the cells, and cell viability and density proved to be completely unaffected by the presence of the metal deposits after 24–48 hours of culture (Supplementary Fig. 4). The adherent cells were transfected with vectors encoding for the proteins of interest and fluorescent tags, and the fluorescent signal in a subset of living cells was acquired by means of low-magnification confocal microscopy using fields of view containing the pattern coordinates. After confocal imaging, the cells were fixed and processed for SEM (see Methods) and, by using the reference markers on the coverslips, we could immediately identify the same subset of cells, acquire higher-resolution SEM images, and analyse all of the acquired data (Fig. 1c).

This method was used to confirm the results of a previous study^14^ of the effects of the overexpression of protein 4.1R (a major structural element of the membrane cytoskeleton)^15^ in HEK cells. Confocal microscopy suggested that 4.1R overexpression led to a number of structural changes: the transfected cells were characterised by a larger surface area and greater filopodia protrusion, a phenotype that was completely reverted when ICln (a multifunctional cytosolic protein)^16^ was overexpressed together with 4.1R (Fig. 2a). The HEK cells were transfected with vectors containing the nucleotide sequence encoding for 4.1R or ICln, and a fluorescent protein (EGFP or DsRed) separated by an IRES sequence (see Methods), and the use of our square-based patterned coverslips allowed us to analyse the same fluorescent cells expressing 4.1R alone (Fig. 2b), ICln alone, or both 4.1R and ICln, which were first identified by means of confocal microscopy and then imaged using the scanning electron microscope (Fig. 2c). The magnification and resolution of the EM consistently revealed a significant increase in cell surface area and filipodia density (with no effect on their length) in the HEK cells overexpressing 4.1R that was absent in the cells co-overexpressing ICln.

In order to demonstrate that our substrates were suitable for the growth and correlative imaging of different cell types, we also performed CLEM experiments using fluorescently labelled HeLa cells (Fig. 3, Supplementary Fig. 5b) and primary rat cortical neurons (Supplementary Fig. 5a).

We here describe the development of a device and protocol that make it possible to perform any kind of correlative imaging experiment involving light and scanning electron microscopy. The main advantages of the method are: 1) the possibility of using substrates of different shapes and sizes with fully customized patterns designed *ad hoc* for specific cell types or objects of interest, and a variety of different metals (titanium, gold, zirconium), thus allowing the greatest experimental flexibility; 2) the possibility of functionalising the surface of the substrates in different ways depending on the cells used (poly-L-lysine, fibronectin, collagen or laminin); and 3) the possibility of using any conventional fluorescence/confocal microscope and scanning electron microscope, thus avoiding the need for expensive integrated microscopes^10^,^11^. Our reference marker system, which can be easily detected by means of both LM and SEM, is simple to create in any laboratory equipped with a conventional metal deposition system simply by depositing one layer of metal on a glass substrate, and allows a region of interest to be tracked when moving from one instrument to the other, even if occasional distortions occur during the processing of electron microscopy samples. In particular, as the entire pattern is visible even to low-magnification SEM, it is possible to zoom in on the appropriate marker and locate the cell of interest in a few seconds. Furthermore, as a coverslip is the general substrate for the SEM imaging of adherent cells, the sample can be conventionally tilted on the SEM stage in order to acquire images from the desired angle of view (Fig. 3c), which allows high-resolution SEM images to be superimposed on 3D reconstructions based on fluorescence images acquired at higher magnification by means of confocal microscopy (Supplementary Fig. 5b). The refractive index of the material of the substrates (glass) perfectly matches the light path of confocal microscopes, and the deposited metals are highly stable under the electron beam of the SEM, thus ensuring best-quality light and scanning electron microscopy images, moreover, surface charge accumulation (after coating and using acceleration voltages ranging from 1 to 5 kV, see Methods) is comparable with that encountered under identical imaging conditions using untreated glass coverslips and silicon (Supplementary Fig. 6). The nature of these materials also guarantees optimal conditions for cell (and primary neuron) growth and viability, and the process of pattern deposition does not contaminate the surface of the substrate with debris as may occur when other approaches are used (e.g. the laser sculpturing of Aclar® film)^7^ (Supplementary Fig. 7). Finally, the use of a patterned substrate does not require any specific software, or holders or stages with fiduciary markers to relocate a sample's position.

For all these reasons, the use of patterned glass coverslips and a robust cell preparation protocol for SEM is a highly promising means of performing rapid, adaptable and accurate correlative experiments.

## Methods

### Pattern design

In order to design the best reference markers for identifying objects of interest by means of both light and scanning electron microscopy, we created different patterns based on the different sizes and morphology of the investigated neurons, HEK and HeLa cells, including one suitable for round fibroblast/epithelial-type cells and another for elongated neuronal-like cells, both of which were designed by generating an AutoCAD® electronic file that could be read by a computerised numerical control (CNC) machine. We started by drawing a single pattern, which was then replicated in order to generate a grid that covered the central area of a 10 mm round glass coverslip (Supplementary Fig. 1), and then replicated the features to fill a square of 10 × 10 cm representing the final stencil mask we wanted to use for metal deposition.

### Stainless steel stencil mask production and maintenance

The mask was produced by Società Italiana per il Chemical Machining (San Donato Milanese, Milan, Italy), which laser cut a 100 μm thick stainless steel substrate with a tolerance of ±0.01 mm. After each use, the mask is thoroughly washed with ethanol (Sigma-Aldrich Co., St. Louis, MO, USA) in an ultrasonic bath (Elmasonic S30H, Singen, Germany) for 10 minutes and dried with nitrogen (N_2_). This cleaning procedure is important to preserve the size of the features and guarantee reproducible electron beam metal deposition.

### Generation of patterned coverslips by means of electron beam deposition

**Figure 1:**
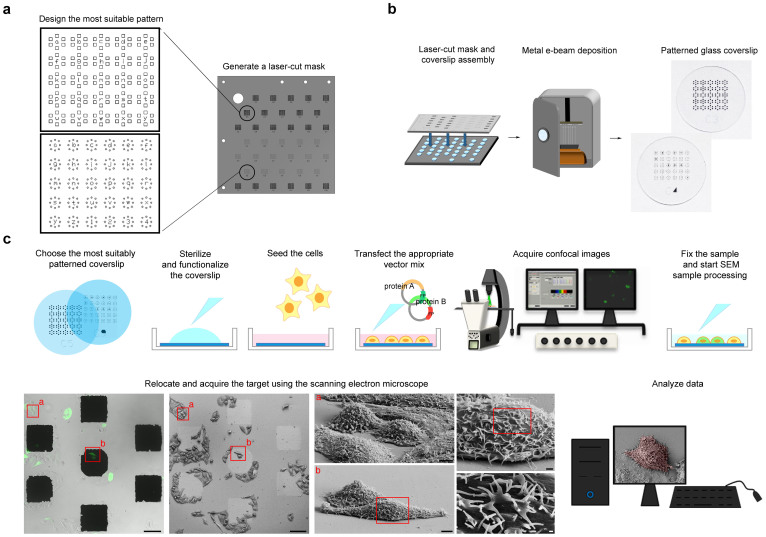
Flow chart showing the process of creating a CLEM patterned glass coverslip and preparing the samples. (a) From pattern design to laser-cut mask. (b) Basic steps in creating a 10 mm diameter patterned glass coverslip. (c) Experimental protocol. The cells are seeded on an appropriately patterned, sterilized and functionalized glass coverslip before being transfected with constructs encoding the investigated and fluorescent proteins. Once the exogenous proteins are adequately expressed, the confocal microscopy images are acquired, and the samples are then fixed, immediately processed for SEM, and transferred to the SEM image acquisition chamber. The acquired images are finally analyzed to obtain the data of interest. Scale bars: 100 μm in the large confocal microscope and SEM large fields of view; 10 μm in the cell zooms: and 1 μm and 200 nm in the details.

Before deposition, glass microscope coverslips with diameters of 10, 13, 15 and 24 mm (VWR International, Milan, Italy) were sonicated (Elmasonic S30H) in ethanol (Sigma-Aldrich Co.) and then in 2-propanol (Sigma-Aldrich Co.) for 30 minutes each, and dried with nitrogen (N_2_). After being put into a coverslip holder with the mask for pattern generation (Fig. 1), they were loaded into a conventional custom-made evaporation system and the pressure inside the deposition chamber was brought to 6.0 × 10^−6^ mbar. The beam was switched on and set to a voltage of 6 kV, and the evaporation was started by gradually increasing the current to 34 mA, a value at which titanium (99.999% pure, Kurt J. Lesker, Hastings, UK) starts to melt, evaporate and deposit at a rate of 0.2 Å/sec. (the same deposition rate was also used for gold and zirconium but, given their different physical properties, it was necessary to use higher currents (respectively 420 mA and 165 mA) and longer deposition times). The film thickness and rate of deposition were controlled by means of a quartz crystal monitor. When the desired thickness was reached (100 nm in the case of titanium), the beam was switched off, the chamber was vented, and the substrates were removed ready to be used for the correlative experiments.

### Sterilisation and functionalisation of the patterned coverslips

Before seeding the cells, the patterned coverslips were sterilized in a stove at 180°C overnight and/or washed in ethanol 70% and/or exposed to UV radiation for 50 minutes, functionalized with a drop of poly-L-lysine solution 0.1% w/v in water (Sigma-Aldrich Co.) for five minutes, and then rinsed with bi-distilled water. For the experiments described in Supplementary Figure 6, the silicon substrates were sonicated in acetone and isopropanol for 10 minutes, and sterilized using ethanol and UV radiation before being functionalized with poly-L-lysine 1 mg/ml.

### Cell cultures

HeLa or human embryonic kidney (HEK) 293T cells were grown in Eagle's minimum essential medium (EMEM; 12-125F, Lonza© Walkersville, Inc., Walkersville, MD, USA) supplemented with 10% fetal bovine serum (FBS), L-glutamine 2 mM, non-essential amino acids 0.1 mM, penicillin 100 U, streptomycin 100 μg/ml, and sodium pyruvate 1 mM, and the cultures were maintained in a humidified incubator at 37°C with 5% CO_2_.

The cells were split every 3–4 days when 80–90% confluent. Briefly, the culture medium was discarded, and the cell layer was rinsed with phosphate buffer saline (PBS: NaCl 136.89 mM, KCl 2.69 mM, Na_2_PO_4_ 1.47 mM, NaOH 10 mM, pH 7.4) to remove all traces of serum, which contains trypsin inhibitor. Trypsin-EDTA 0.25% was added, and the culture dish was put in an incubator at 37°C for five minutes, after which complete growth medium was added and the cells were dissociated by means of gentle pipetting. Appropriate aliquots of the cell suspension was added to new culture dishes, and the cultures were incubated again at 37°C.

For the CLEM experiments, HeLa or HEK cells were seeded at a concentration of 160 cells/mm^2^ on the patterned coverslips functionalized with poly-L-lysine, and the cultures were incubated at 37°C until cell adhesion.

After the patterned coverslips were cleaned with 100% ethanol at 37° for two hours, washed with sterilized H_2_O, heated at 180°C for five hours, functionalized with poly-L-lysine 1 mg/mL, and rinsed with water, rat hippocampal neurons were seeded on the substrates at a concentration of 160 cells/mm^2^ in Dulbecco's modified Eagle's medium (DMEM) supplemented with 10% FBS, L-glutamine 2 mM, non-essential amino acids 0.1 mM, penicillin 100 U, streptomycin 100 μg/ml, and sodium pyruvate 1 mM (all of the reagents were purchased from Life Technologies™, Monza, Italy).

### Plasmids

The 4.1R-IRES-GFP and ICln-IRES-dsRED vectors^14^, expressing the 4.1R135 or ICln proteins and the fluorescent protein as two distinct polypeptides, were a kind gift of Dr. Claudia Bazzini (Univerity of Milan, Milan, Italy) and of Prof. Markus Paulmichl (Paracelsus Medical University, Salzburg, Austria), respectively. The empty vectors: pIRES2-EGFP and pIRES2-dsREDexpress were used as controls.

HEK and HeLa cells were transiently transfected 24 hours post-seeding. In the co-transfection experiments, each vector was equimolar in the transfection mix.

Neurons were transfected with pEYFP-C1 (Clontech Laboratories, Inc.© - St. Germain en Laye, France).

### Transient transfection

The cell lines were transiently transfected using the calcium phosphate method, adding 1 μg DNA and 2.5 μl CaCl_2_ 2.5 M (Sigma-Aldrich Co.) for each 1.6 cm dish, and distilled water to bring the total volume to 25 μl. After five minutes, 25 μl of HBS 2× (NaCl 140 mM, NaHPO_4_ 1.5 mM, HEPES 50 mM, pH 7.05) were gently added to the CaCl_2_/DNA mix, and the transfection mixture was then added dropwise to the cell culture. The dish was transferred to the incubator at 37°C and 5% CO_2_ and, after 6–10 hours, the medium was replaced by warm complete medium and incubation resumed. The neurons were transfected after three days *in vitro* (DIV) using 3 μg of DNA and the same procedure as that described for the cell lines, and imaged on *in vitro* day 5.

### MTT assay

HeLa cells were seeded at a concentration of 600 cells/mm^2^ on 10 mm glass coverslips or patterned substrates that were sterilized with 70% ethanol and 50 minutes of UV radiation, and functionalized with poly-L-lysine 1 mg/mL. Twenty-four and 48 hours after seeding, the cells were incubated for four hours with the MTT solution (3-(4,5-dimethylthiazol-2-yl)-2,5-diphenyltetrazolium bromide, Cell Proliferation Kit I, Cat. No. 11465007001, Roche Diagnostic GmbH, Mannheim, Germany). After incubation, the purple formazan salt crystals were solubilised by adding the solubilisation solution and incubating the plates overnight in a humidified atmosphere (37°C, 5% CO_2_). The solubilised formazan product was spectrophotometrically quantified using an ELISA reader (Tecan Sunrise, Tecan Group Ltd., Männedorf, Switzerland). Cell viability was evaluated at each time point in four independent experiments for each substrate.

### Confocal microscopy

Images of the cells on the patterned coverslips were acquired using a Leica TCS SP5 confocal inverted microscope (Leica Microsystems GmbH, Wetzlar, Germany) with an HC PL FLUOTAR 20× 0.5 (NA 0.5) objective, a pixel size of 378.8 × 378.8 nm, and a scan speed of 400 Hz (low resolution/low magnification). EGFP was excited with a 488 nm laser line, and the PMT1 emission bandwidth was 500–550 nm; DsRED was excited with a 561 nm laser line, and the PMT2 emission bandwidth was 570–650 nm; EYFP was excited with a 514 nm laser line, and the PMT2 emission bandwidth was 525–600 nm; and the Hoechst nuclear dye was excited with a 405 nm laser line, and the PMT1 emission bandwidth was 415–455 nm. Bright field images were also acquired with the fluorescence channels superimposed (ImageJ 1.45 software, Wayne Rasband, NIH, USA) in order to visualise the pattern together with the transfected and non-transfected cells on the coverslip. During confocal image acquisition, the cells were kept alive in the microscope incubator (Okolab, Naples, Italy) at 37°C and 5% CO_2_ in DPBS (PBS supplemented with CaCl_2_ 1 mM, MgCl_2_ 0.5 mM, and glucose 25 mM, pH 7.4).

The high-resolution images for the 3D reconstruction were acquired by means of an HCX PL APO 63× 1.4 (NA 1.4) objective, a pixel size of 98.8 × 98.8 nm, and a z-step size of 250 nm using the same parameters as those described above. The 3D images were produced using the UCSF Chimera package from the Resource for Biocomputing, Visualization and Informatics at the University of California, San Francisco (supported by NIH P41 RR-01081) (Supplementary reference).

### Cell adhesion analysis

HeLa cells were seeded at a concentration of 600 cells/mm^2^ on 10 mm glass coverslips or patterned substrates that were sterilized with 70% ethanol and 50 minutes of UV radiation, and functionalized with poly-L-lysine 1 mg/ml. Twenty-four and 48 hours after seeding, the cells were fixed with paraformaldehyde 4% in PBS for 30 minutes, rinsed with PBS, incubated with Hoechst 33342 0.01 mg/mL (H1399, Molecular Probes®, Life Technologies Europe BV, Monza, Italy) for 30 minutes, and mounted in 90% glycerol. The density of the cells grown on the substrates was evaluated at each time point in four independent experiments for each substrate by counting the density of cell nuclei in fields of view (775 × 775 μm) acquired with the 20× objective of the confocal microscope using ImageJ software. Both bright field and fluorescence images were recorded.

### SEM sample preparation

The cells were processed for SEM imaging immediately after the acquisition of the confocal images. They were fixed with glutaraldheyde 1.2% in NaCacodylate 0.1 M for one hour, washed three times with NaCacodylate 0.1 M for 10 minutes, and post-fixed with osmium tetroxide (OsO_4_) 1% in NaCacodylate 0.1 M for one hour. After removing the OsO_4_ solution and rinsing twice with bi-distilled water, the samples were gradually dehydrated by means of an ethanol series, and then dried using an Emitech K850 critical point drier (Bad Schwalbach, Germany) or hexamethyldisilazane (HMDS). All of the reagents were purchased from Electron Microscopy Sciences (EMS, Hatfield, PA, USA). Once dried, the samples were sputtered with gold (Polaron E5100 Sputter Coater, Bad Schwalbach, Germany) and the images were acquired at 1–5 kV using a field emission gun scanning electron microscope (Ξigma, Zeiss, Oberkochen, Germany) with a secondary electron detector (SE1). The samples for the experiments described in Supplementary Figure 6 were imaged before and after gold coating as described above.

### Generation of the Aclar® patterned substrate for CLEM

A pattern with coordinates consisting of an asymmetrical mesh of squares of about 140 μm was sculpted on Aclar® film using the pulsed laser of a microdissecting microscope^7^ (Leica Microsystems GmbH, Wetzlar, Germany). Before seeding the cells, each 10 mm Aclar® disc was sterilized using ethanol 70% and UV radiation for 50 minutes, and then functionalized with poly-L-lysine. The cells were cultured and transfected as decribed above.

## Author Contributions

L.B. designed and carried out the cell cultures and CLEM protocols, and co-wrote the manuscript; E.S. designed and produced the patterned substrates, and co-wrote the manuscript; S.R. designed the fluorescence experiments and contributed to the pattern design; D.M. acquired the scanning electron microscopy images; P.M. supervised the project; and M.F. supervised the project and co-wrote the manuscript.

## Supplementary Material

Supplementary Information supplementary figures

## Figures and Tables

**Figure 2:**
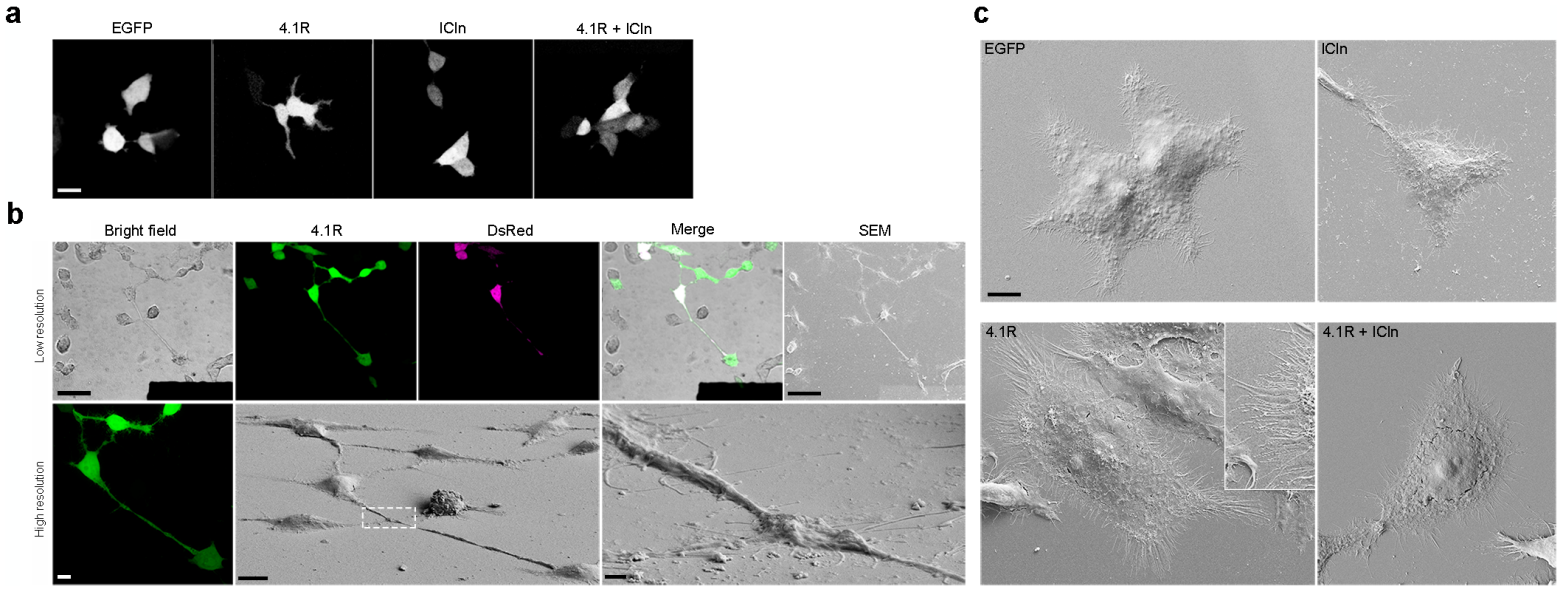
4.1R expression increases cell area and the number of filopodia protruding from cell edges. (a) Confocal images showing the morphology of HEK cells overexpressing the indicated proteins (only the EGFP channel is shown). The area of the cells expressing 4.1R alone is larger than that of the cells expressing EGFP or ICln alone, or those expressing both 4.1R and Icln. Scale bar: 20 μm. (b) CLEM was used to characterise the effect of 4.1R overexpression on cell morphology precisely. The HEK cells adhering to the patterned coverslips were transfected with vectors encoding 4.1R-IRES-EGFP (4.1R) and soluble DsRed (DsRed) (see On-line Methods). The cells expressing the protein of interest were identified in low magnification confocal microscopy fields of view in bright field and fluorescence imaging mode (Merge, upper panel: scale bar 50 μm). High-resolution z-stacks of the selected cells were acquired in order to characterise morphology in living cells (maximum projection shown in left lower panel: scale bar 10 μm). The cells were then processed for SEM, the same field of view was rapidly relocated, and the same cells were imaged at low magnification (right upper panel: scale bar 50 μm), and then high-magnification images were acquired in order to characterise the selected cells morphologically at nanometre resolution (lower panel: scale bar 10 μm; inset 1 μm). (c) Representative SEM images of HEK cells expressing the indicated protein. The overexpression of 4.1R alone (but not the co-overexpression of 4.1R and ICln) increased cell area, as well as the number and density of filopodia. Scale bar 10 μm.

**Figure 3:**
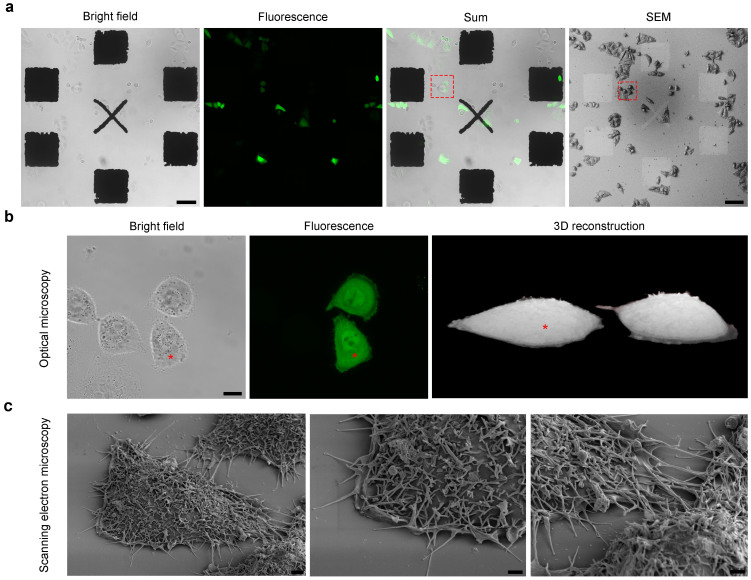
High-resolution optical and electron microscopy images of HeLa cells expressing EGFP. (a) Fields of view showing HeLa cells overexpressing soluble EGFP growing on the patterned coverslip as acquired by means of optical microscopy (Bright field, Fluorescence and Sum) and SEM. Scale bars 100 μm. The pattern was developed so that it could be easily visualised by both microscopes in order to allow the rapid relocation of the same cells when moving from one to the other. (b) High-resolution optical images (Bright field and Fluorescence maximum projections), and a 3D reconstruction of the two EGFP-expressing cells (red box) in (a). Scale bar: 10 μm. (c) Scanning electron microscopy images of the EGFP-expressing HeLa cell asterisked in (b) showing the highly preserved ultrastructure obtained after SEM preparation on the patterned substrates, and the high-resolution details observable after tilting the sample holder. Scale bars 2 μm.
