# Supplementary material for: Customized patterned substrates for highly versatile correlative light-scanning electron microscopy
Source: Sci Rep. 2014 Nov 13;4:7033. doi: 10.1038/srep07033 (PMC4229662; doi:10.1038/srep07033)
Supplement: Supplementary Information — supplementary figures [file srep07033-s1.pdf]

CUSTOMIZED PATTERNED SUBSTRATES FOR HIGHLY VERSATILE CORRELATIVE LIGHT-SCANNING ELECTRON MICROSCOPY

Lorena Benedetti[1,2]*, Elisa Sogne[1,3,4]*, Simona Rodighiero[1], Davide Marchesi[1], Paolo Milani[1,3], Maura Francolini[1,2]

[1]Fondazione Filarete for Biosciences and Innovation, Viale Ortles 22/4, 20139, Milan, Italy
[2]Department of Medical Biotechnology and Translational Medicine, Università degli Studi di Milano, and National Research Council (CNR) Neuroscience Institute, Via Vanvitelli 32, 20129 Milan, Italy
[3]Interdisciplinary Centre for Nanostructured Materials and Interfaces (CIMaINa), and Department of Physics, Università degli Studi di Milano, Via Celoria 16, 20133 Milano, Italy
[4]European School of Molecular Medicine (SEMM), IFOM-IEO, Via Adamello 16, 20139 Milano, Italy

***These authors contributed equally to this work**

Correspondence and requests for materials should be addressed to M.F. (maura.francolini@fondazionefilarete.com, maura.francolini@unimi.it)

SUPPLEMENTARY FIGURES

Figure S1

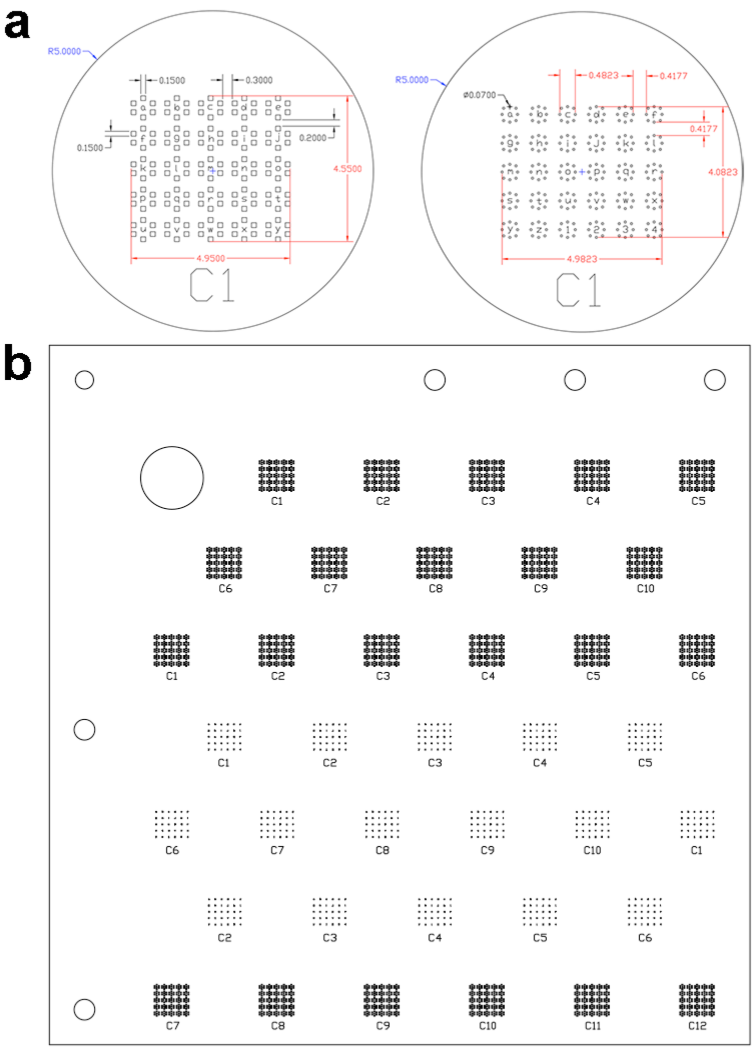

Figure S1: AutoCAD® drawing of patterns and mask.

(a) Left. Drawing of a square-based pattern: each basic element consists of six 150 x 150 µm squares surrounding a letter and is repeated 25 times (5 rows x 5 columns) to create a finding pattern. Right. Drawing of a circle-based pattern consisting of eight 70 µm diameter solid circles surrounding a letter or number, which is repeated 30 times (5 rows x 6 columns). All of the measurements are in mm. (**b**) Drawing of 10 x 10 cm stainless-steel stencil mask with the square- or circle-based layout repeated respectively 22 and 16 times.

Figure S2

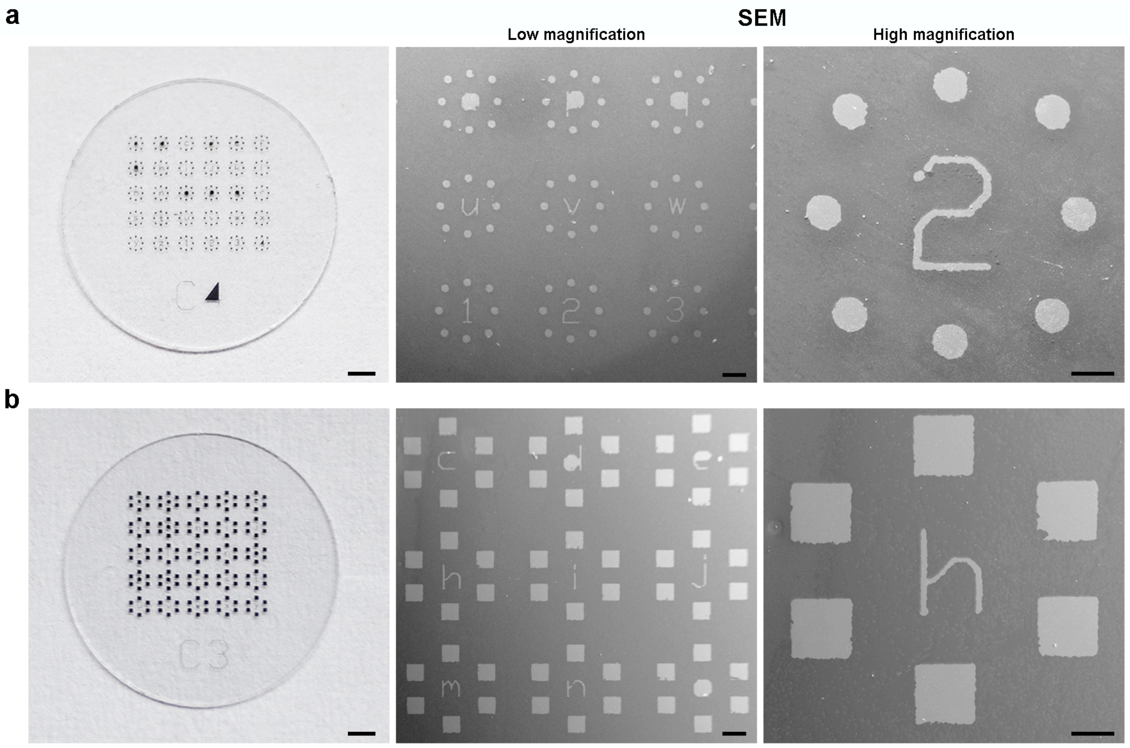

Figure S2: SEM visualization of the customized patterned glass coverslip

(**a**) Circle-based pattern deposited on a 10 mm glass coverslip (scale bar 1 mm); pattern detail acquired by SEM (scale bar 200 µm); zoom of a single pattern on the same glass coverslip (scale bar 100 µm). (**b**) Square-based pattern deposited on a 10 mm glass coverslip (scale bar 1 mm); pattern detail acquired by SEM (scale bar 200 µm); zoom of a single pattern on the same glass coverslip (scale bar 100 µm).

Figure S3

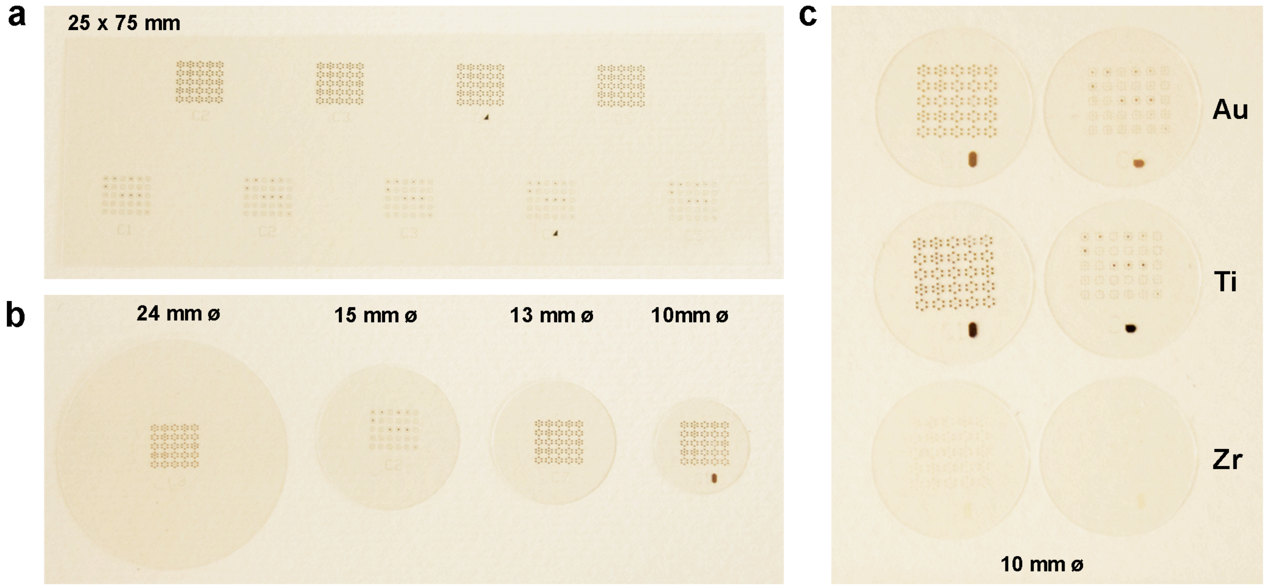

Figure S3: Representative examples of differently patterned substrates.

(**a**) A 25 mm x 75 mm glass slide on which nine different reference grids have been deposited: top) four patterns designed for round/polygonal cell types; bottom) five patterns designed for elongated/neuronal cell types. (**b**) Four coverslips of respectively 24 mm, 15 mm, 13 mm and 10 mm deposited with a pattern of 70 nm thick gold. (**c**) Ten-millimetre coverslips with square- (left) and circle-based (right) patterns of gold, titanium and zirconium (from top to bottom).

Figure S4

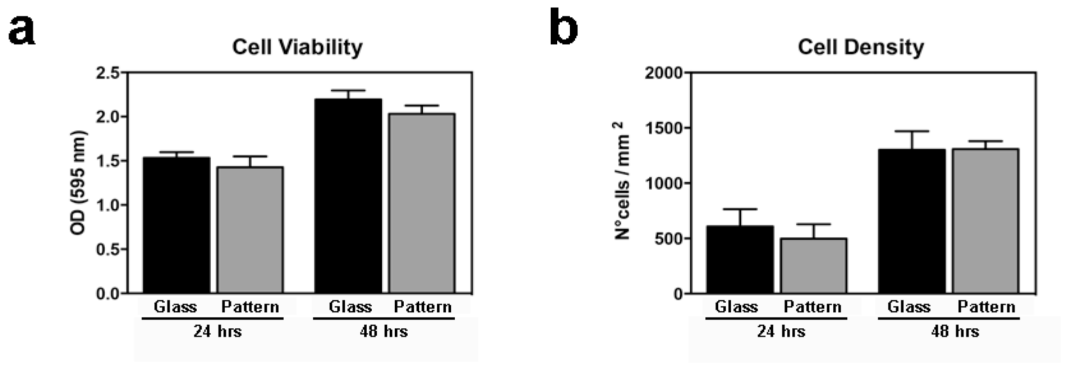

Figure S4: Cell viability and density after seeding on glass or patterned substrates

(**a**) Cell viability was measured by means of MTT assays 24 and 48 hours after seeding on glass coverslips and patterned glass substrates; there was no significant difference in cell viability at either time point regardless of the substrate. (b) The density of HeLa cells adhering to the glass coverslips or patterned glass substrates remained unchanged 24 and 48 hours after seeding. The statistical analyses were made using GraphPad Prism® version 6.0c and Student's t test. Mean values were considered statistically different when $p<0.05$.

Figure S5

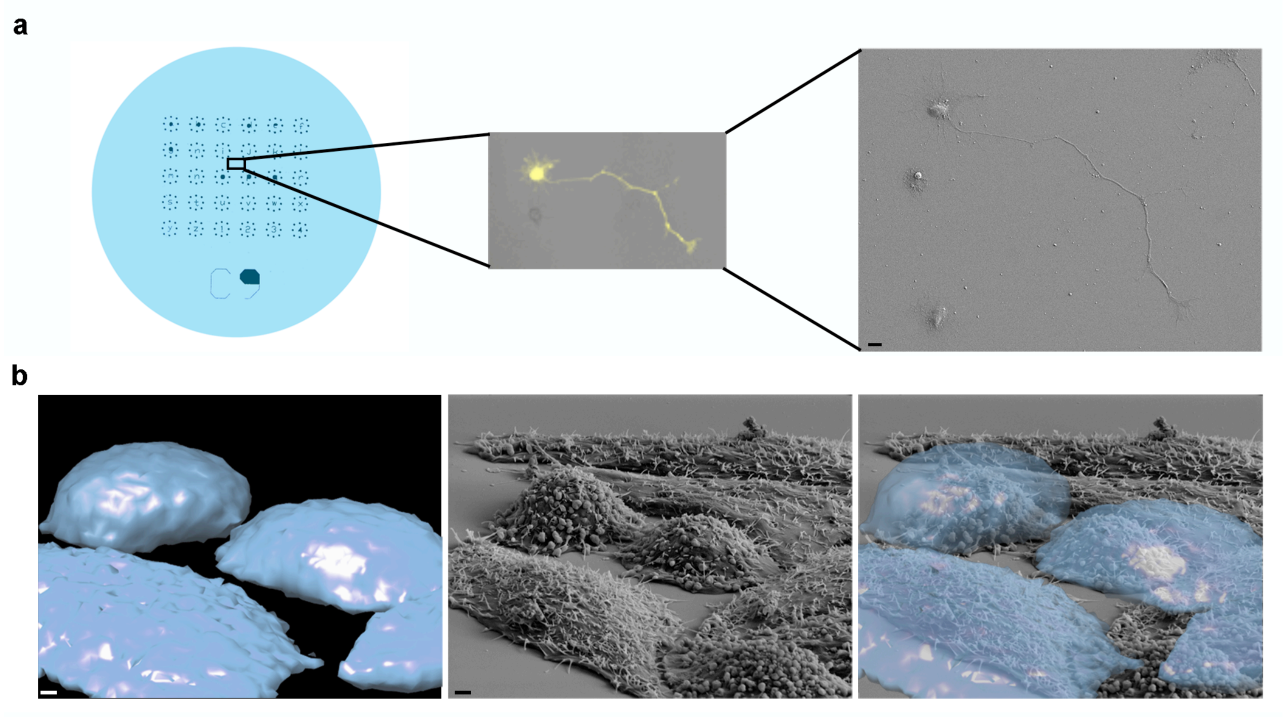

Figure S5: CLEM experiment using primary rat cortical neurons and an overlay of 3D-reconstructed EGFP-overexpressing HeLa cells with tilted SEM image.

(**a**) SEM image (scale bar 10 µm) and low-resolution confocal image of the same primary rat cortical neuron expressing EYFP before and after processing for SEM. Neurons were grown on glass coverslips patterned using the round marker reference system.

(**b**) 3D reconstruction of a high-resolution fluorescence confocal z-stack covering the entire volume of EGFP-overexpressing HeLa cells obtained using Chimera software (Pettersen et al., 2004) aligned with a scanning electron microscope tilted image of the same cells acquired after CLEM sample preparation. Scale bars 2 µm.

Figure S6

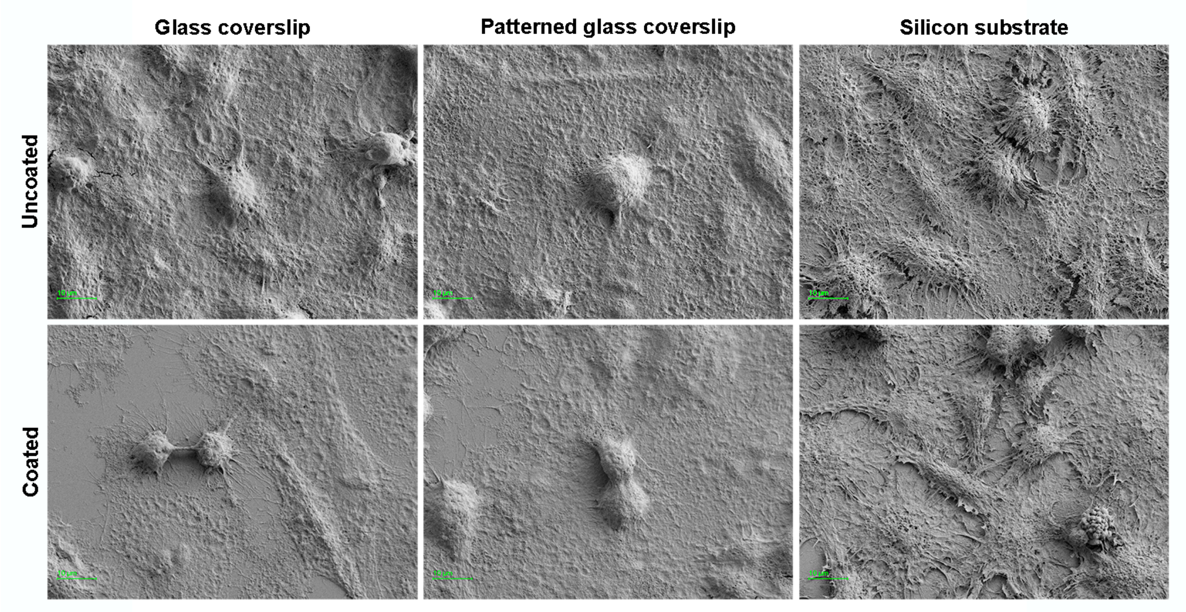

Figure S6: SEM images of cells grown on glass coverslips, patterned glass substrates and silicon substrates with and without gold coating.

SEM images of HeLa cells cultured on uncoated (upper panel) or gold coated (bottom panel) glass coverslips, glass patterned coverslips and silicon substrates acquired with the same acceleration voltage (1kV) and secondary electrons detector (SE1) (see Methods). With 1kV acceleration voltage, no surface charging is observed on samples regardless of the type of substrate and of coating. At higher acceleration voltages (2 to 5 kV), uncoated cells grown on glass coverslips (with and without pattern) but not on silicon substrates, accumulate surface charges. At this voltages, charge accumulation never occurs on gold coated samples (not shown).

Figure S7

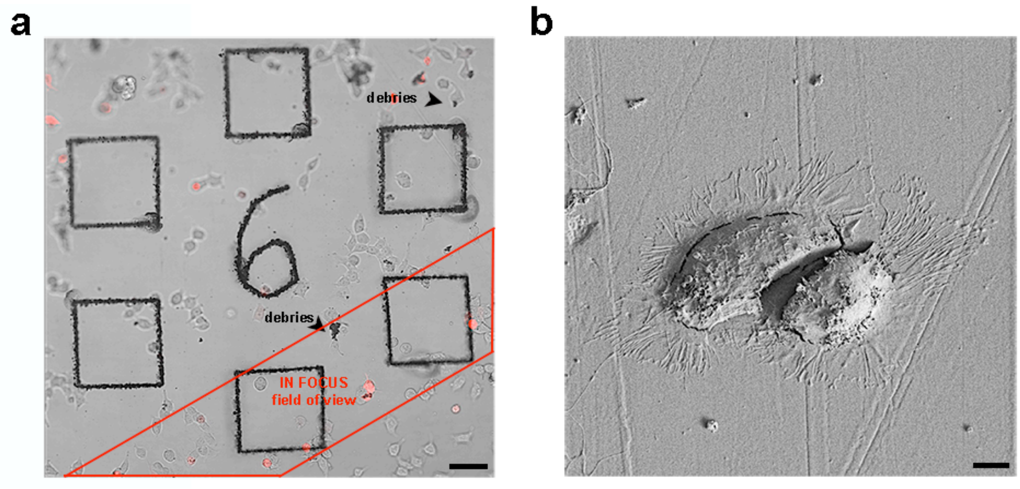

Figure S7: Correlative experiments on patterned Aclar® films.

(**a**) Sum of low-resolution, bright field and fluorescence images of fluorescently labelled cells grown on patterned Aclar® discs. Pulsed laser beam pattern generation on Aclar films is time-consuming and produces debris; furthermore, the flexibility of the polymer means that only a small area of the patterned film is in focus (red box). Scale bar 50 µm. (**b**) SEM image of HEK cells cultured on Aclar® film: the flexibility of the substrate can cause damage and artifacts that alter the structure the cells, thus hampering the precise quantitative analysis of cell morphology in CLEM experiments. Scale bar 5 µm.

SUPPLEMENTARY REFERENCE

Pettersen, E.F., Goddard, T.D., Huang, C.C., Couch, G.S., Greenblatt, D.M., Meng, E.C., and Ferrin, T.E. "UCSF Chimera - A Visualization System for Exploratory Research and Analysis." *J. Comput. Chem.* **25**(13):1605-1612 (2004).
